# Donor-Derived Tuberculosis: A Case Report and the Role of Communication Gaps in Transplantation Safety

**DOI:** 10.1155/2021/8816426

**Published:** 2021-04-17

**Authors:** Wanessa T. Clemente, Luciana C. Faria, Gláucia F. Cota, Leandro Ricardo de Navarro Amado, Jaqueline G. F. Oliveira, Silvana Spíndola de Miranda, Omar Lopes Cançado, Roberta M. C. Romanelli, Agnaldo S. Lima, Luiza Bastos Frade, Fernando Lucas, Marcelo Dias Sanches

**Affiliations:** ^1^Alfa Institute of Gastroenterology - Liver Transplant Program, Hospital das Clínicas/UFMG, Brazil; ^2^Laboratory Medicine Department, Faculty of Medicine, Federal University of Minas Gerais (UFMG), Brazil; ^3^Internal Medicine Department, Faculty of Medicine, Federal University of Minas Gerais (UFMG), Brazil; ^4^Transplant Program, Hospital das Clínicas/UFMG, Centro de Pesquisas René Rachou, Fundação Oswaldo Cruz, FIOCRUZ, Brazil; ^5^OPO Transplant Center, MG Transplante, Belo Horizonte, Brazil; ^6^Department of Paediatrics, Faculty of Medicine, Federal University of Minas Gerais, Brazil; ^7^Kidney Transplant Unit, Hospital das Clínicas/UFMG, Brazil; ^8^Surgery Department, Faculty of Medicine, Federal University of Minas Gerais, Brazil; ^9^Alfa Institute of Gastroenterology - Pancreas Transplant Program, Hospital das Clínicas/UFMG, Brazil

## Abstract

Donor-derived tuberculosis (DD-TB) accounts for less than 5% of TB cases and is considered a rare event. In the transplant setting, the frequency of active TB is estimated to be 20 to 74 times higher than that in the general population, and it is associated with high mortality. In this context, the main strategy to minimize the risk of DD transmission is to identify high-risk donors. Despite screening recommendations, failures may result in a breakdown of safety that ends in the transmission of potentially fatal diseases. This report describes a case of DD-TB and emphasizes communication gaps that may occur between organ procurement organizations and transplant centers. Failure in reporting results, lack of exchanging information regarding recipients from the same donor, and inefficient communication between organ procurement organizations and transplant centers are lacks that may be prevented by a more efficient approach towards screening protocols and communication.

## 1. Introduction

Most tuberculosis (TB) cases in solid organ transplant (SOT) recipients are caused by the reactivation of latent tuberculosis infection (LTBI), and only 4% are considered donor-derived [1–3]. Active TB in the donor is recognized as an unacceptable risk and contraindicates organ donation [4–6]. However, current screening protocols for deceased donors are usually based on chest X-ray findings (which may be nonspecific), epidemiological risks, and previous TB history. Regrettably, these features may not identify extrapulmonary and disseminated TB. Therefore, TB recognition is not always feasible, particularly when unusual symptoms are under differential diagnostic consideration and the potential donor has low-epidemiological risk [7–10]. Thus, unfortunately, despite organ procurement protocols, pretransplantation screening fails to identify TB in the donor in many cases [7, 11]. Even when these cases are identified, communication gaps may result in donor-derived infections (DDI), which may be associated with death or poor outcomes [10, 12–14].

This report describes a case of unrecognized disseminated TB in the donor with a devastating outcome for organ recipients. The donor was a pregnant woman who died soon after delivery due to TB involvement of the central nervous system (CNS) and lungs. The diagnosis was retrospectively established by mycobacterial culture of the respiratory sample (positive result obtained two months after donor death) and the diagnosis of congenital TB in her baby. Additionally, this report also highlights the need for a data bank and donor sample analysis to trace infections and reinforces the importance of better communication between transplantation teams and organ-harvesting centers [12].

### 1.1. The Index Case - Simultaneous Pancreas Kidney Recipient

A 45-year-old male received a simultaneous pancreas kidney (SPK) transplant due to diabetes and end-stage renal disease (ESRD) . After transplantation, he had no major clinical complications and was discharged on postoperative day 30. His previous tuberculin skin test (TST) was negative, and he denied known TB exposure. The immunosuppressive regimen included basiliximab, prednisone, tacrolimus, and mycofenolate mofetil. During the second month posttransplant, he returned to the hospital complaining of fever, night sweats, and chills. Abdominal ultrasonography revealed perigraft collections (renal and pancreatic abscesses), whereas the chest radiograph was normal. He received broad-spectrum antibacterial treatment and underwent percutaneous drainage of the abscesses, with transient resolution of fever. Laboratorial analysis revealed acid-fast bacilli (AFB) on Ziehl-Neelsen stain. Antituberculous therapy was started with standard drugs: rifampin, isoniazid, pyrazinamide, and ethambutol

**Figure 1:**
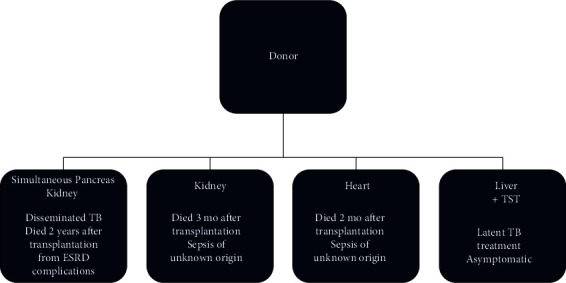
Diagram showing the evolution of patients who received organs from the donor with tuberculosis. ^∗^SPK recipient. Outcome: cured from tuberculosis. Death related to end-stage renal disease (ESRD) 2 years after transplantation.

Concurrently, considering that the recipient's TB abscesses were located near the grafts, suggesting donor involvement, the transplant harvesting center was contacted for additional information regarding the donor and the other organ recipients. At that time, two recipients had already died, and a look-back investigation was carried out (Figure 1).

**Figure 2:**
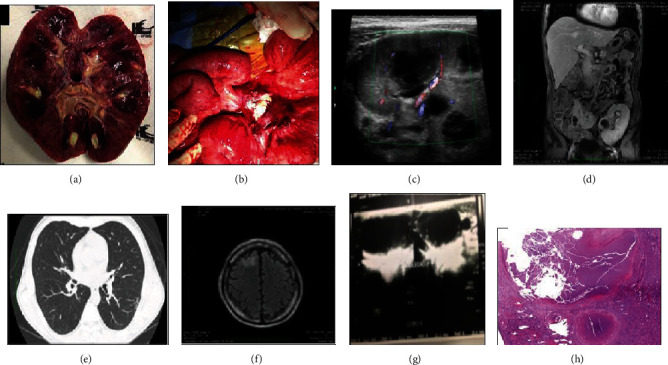
Images, surgical findings, and histological exams of the SPK transplant recipient: (a) caseous necrosis in the explanted kidney; (b) caseous necrosis in the mesentery; (c) nodule in the renal hilum, diffuse urothelial thickening, and compression of renal vein; (d) pancreas: collection adjacent to pancreatic graft; (e) lung endobronchial spread: “tree-in-bud”; (f) brain abscess: frontal lesion with perilesional halo of edema; (g) thyroid: increased volume, heterogeneous collection on the left lobe; (h) caseating granuloma in renal biopsy.

The patient subsequently presented with anti-TB drug toxicities: haemolytic anaemia (related to rifampicin) and blurred vision (due to ethambutol), both in the 2nd month of treatment resulting in a change of therapy. At this time, the patient presented disseminated disease involving grafts, lungs, CNS, and thyroid.The clinical deterioration of the patient imposed immunosuppressive cessation, leading to acute cellular rejection of the grafts, and dual graft loss with return to hemodialysis and insulin therapy. The patient underwent exploratory laparotomy with a surgical finding of caseating necrosis all over the mesenterium and around pancreatic graft, but affecting the renal graft. The removal of the renal graft was the only viable treatment encountered (Figure 2). After several ultrasound guided punctures to drain intra-abdominal TB abscesses and 18 months of anti-TB therapy, the patient was considered cured. However, he died from complications related to ESRD and dialysis, two years after transplantation.

### 1.2. The Donor

The donor was a 23-year-old, 36-week pregnant woman with a history of intense headache who was admitted to the hospital emergency room, and within 72 hours of admission, she developed mild fever, neck stiffness, and seizures. The cerebrospinal fluid (CSF) was considered abnormal with increased cellularity and protein. Although no microorganisms were identified in the CSF and blood, empirical treatment for meningoencephalitis was started 72 hours after hospital admission (ceftriaxone followed by anti-TB standard treatment and acyclovir), and other culture samples were collected. Respiratory secretions were negative for acid-fast bacilli (AFB) stain. Her level of consciousness decreased, and she underwent an emergency caesarean section on the 6th day of admission. Brain computed tomography (CT) scans performed after the procedure revealed diffuse subarachnoid bleeding. The patient was diagnosed with brain death (day 9) and had elected to be an organ donor, considering as primary diagnosis, CNS bleeding. Nearly two months after her death, the tracheal aspirate culture became positive for *M. tuberculosis*. At that time (2 months after delivery), her child presented with fever of unknown origin and was admitted to the same hospital, being diagnosed with disseminated TB. The *M. tuberculosis* strain isolated from the child presented no resistance to first-line anti-TB drugs, and he was discharged home after seven days of standard TB treatment.

### 1.3. The Liver Recipient

The liver recipient was a 55-year-old man diagnosed with Caroli disease who was transplanted because of recurrent bacterial cholangitis. Due to a positive TST before surgery, he was treated for LTBI with isoniazid, soon after the transplant. The treatment was maintained for six months, and the patient has not developed any manifestations compatible with active TB to date.

### 1.4. The Heart Recipient

The heart recipient was a 40-year-old woman diagnosed with Chagas cardiomyopathy who underwent a heart transplant for class IV heart failure. Three months after the procedure, she was admitted to the hospital with fever and malaise. Within three days after admission, she developed severe septic shock and died despite broad-spectrum antibiotic therapy. No bacteria or fungi were identified in cultures (blood, urine, and tracheal aspirate samples); however, no specific mycobacterial direct exam or culture was requested. Although the patient did not receive a specific diagnosis, her chest CT revealed diffuse pulmonary involvement.

### 1.5. The Other Kidney Recipient

The kidney recipient was a 45-year-old man with systemic hypertension and terminal hypertensive nephropathy. During the second month after transplantation, he developed mesangioproliferative glomerulonephritis and graft loss and returned to dialysis. Three months after transplantation, he returned to the hospital with sepsis of unknown origin and died despite antimicrobial therapy, without isolation of any specific infectious agent. Similar to the heart recipient, no specific test for mycobacteria was requested.

Thereby, the TB diagnosis contributed to the fatal outcome in one patient and may be the cause of infectious disease complication after transplantation in the other two. Unfortunately, no autopsy was performed on the deceased organ recipients, and therefore, for both the heart and kidney recipients, the TB diagnosis is only presumptive.

## 2. Discussion

Transplantation is the treatment of choice for some types of end-stage organ failure. However, transplantation has risks related to the procedure itself and due to immunosuppression. Still, there are several holdups associated with care, including the lack of important and complete information concerning donor screening, which can potentially lead to a notable reduced quality of life or may even cause death [15].

Infections represent a major cause of morbidity and mortality for SOT recipients. Risk level (RL) classification regarding DDI transmission involves the use of a grading system to rank recommendations, resulting in classifications that vary from a standard risk to an unacceptable risk [16]. In this regard, active TB is an absolute contraindication for organ donation. Unfortunately, even active TB can be overlooked and therefore mistaken for other diseases [10].

This case report calls attention to the importance of careful donor selection to reduce the risk of transmission of potentially lethal but treatable diseases. Typically, DD-TB is commonly related to the donor's epidemiology and clinical history, even if TB is not initially recognized [3, 9, 10, 17, 18].

In this case, the donor had an unrecognized disseminated TB, with no history of previous TB or exposure and, except for pregnancy, no known immunosuppressive conditions. Immune modulation in pregnancy occurs due to hormones and dysfunction in the lymphocyte activity [19]. The local TB prevalence for the donor's area of residence is approximately 30 cases/100,000 inhabitants, representing an intermediate risk [2, 3, 20] (access March 2021 https://www.saude.mg.gov.br/tuberculose).

Specific policies may be established to improve the recognition of the disease in donors [4]. Several transplant scientific societies developed guidelines to assist in the screening of potential organ donors and recipients [6, 10, 21–25]. However, because these recommendations are not mandatory for all scenarios of practice, they are therefore not always incorporated into OPO standard procedures. For risk factor assessment, OPOs should obtain a donor history of symptoms consistent with active TB, as past diagnosis of TB infection (active or latent), homelessness, alcohol abuse or injection drug use, incarceration, recent exposure to persons with active TB, or travel to areas where TB is endemic. When risk factors are identified, further testing and radiological assessments are warranted. Notwithstanding, active TB is a well-known contraindication to organ donation [4, 6, 26–28].

In this reported case, the donor had no detectable abnormalities on the chest X-ray and AFB-negative smears, whereas the culture became positive two months after procurement. Although sputum culture has higher sensitivity than AFB [29], the time to positivity ranges from weeks to months [29–32]. In this situation, molecular tests present greater sensitivity and specificity but are done when pulmonary TB is suspected [30, 31]. The donor had no respiratory symptoms or compatible image, and the AFB was negative, and therefore, TB was not suspected. However, the CSF analysis showed an elevated protein level with pleocytosis. Subsequently, the patient continued to decline neurologically, and brain death occurred nine days postadmission. The intracerebral bleeding was likely secondary to cerebral vasculitis due to TB infection, in a retrospective analysis. According to current recommendations, in cases of donor death due to meningoencephalitis (ME) without a proven cause, the donation should be avoided [4]. Additionally, to improve screening strategies, certain potential findings should be scrutinised such as the existence of any comorbidity that may support stroke, the presence of fever at illness presentation/admission in the potential donor, CT/MRI scan of the head, or CSF findings consistent with an infectious process, and whether the donor was an immunosuppressed host or had any potential environmental exposures associated with organisms causing ME [4]. In this case, donor cause of death was considered CNS bleeding, and unfortunately, the same occurred in many other reported cases, resulting in DD-TB [7, 9, 10, 14, 33, 34]. Worth mentioning that the diagnosis of tuberculous meningitis is challenging, and the available microbiological tests fail to attain the accuracy standards required, with poor sensitivity and delayed results.

DD-TB is considered proven if donor and recipient isolates were reported to be identical or clonal through molecular analysis. In the absence of definitive confirmation of similar isolates, DD-TB is classified as probable if there is a suspected transmission and TB was identified in multiple recipients of one donor, or if donor and recipient shared more than one epidemiologic or clinical feature (e.g., TB diagnosis in a donor plus TB in the recipient early posttransplantation) or possible, if there is a suspected transmission event but the only criterion met is a donor risk factor for TB (e.g., donor residence in TB endemic area and absence of recipient risk factors for TB) [10].

The major limitation to confirm DDI is to have a positive donor sample and genetic sequencing of the pathogen matching donor and recipient samples. Usually, there is often a time gap between the donation and the development of the disease. In this reported case, TB was confirmed in a donor sample two months after donation, and the result was still not properly informed because there was no tracking system to request the pending microbiological results. As such, donor transmission is usually considered probable or possible, depending on the data available, but is much less often confirmed [35].

Despite the fact that it is desirable to have molecular typing and analysis of patterns of the isolates, this is not always possible. Frequently, samples have sometimes been disposed of at the time of diagnosis, considering that *M*. *tuberculosis* is only identified in culture after 4 to 6 weeks. In this case, we had microbiological confirmation by a positive acid-fast bacillus (AFB) and culture on samples of the donor and a positive mycobacterial culture on the sample of the recipient. However, the donor isolate was not available when the diagnosis of the recipient was established. Therefore, in the absence of isolates to proceed with fingerprinting, the epidemiological link between the donor and recipient was based on early TB onset in the posttransplant period [2]. Anyhow, it is important to emphasize that the graft was the first topography for TB diagnosis in the recipient, a clear indication of donor transmission.

Here, the liver transplant recipient was treated for latent TB and did not develop the disease. The two other organ recipients from the same donor presented fever of unknown origin, sepsis, and organ dysfunction a few months after transplantation. These findings could be compatible with TB donor transmission [3, 36, 37], but the transmission was confirmed only for the SPK transplant recipient.

As previously mentioned, DD-TB is difficult to recognize in both donor and recipient. A recent review retrieved 36 cases of proven (*n* = 17), probable (*n* = 8), and possible (*n* = 11) DD-TB among 16 lung, 13 kidney, 6 liver, and 1 heart recipients. The median time to clinical presentation or diagnosis was 2.7 months, and fever was the most frequent presenting symptom. Allograft involvement was common. Graft loss occurred in ~20% of patients. All-cause mortality was 25% [10]. As pointed out in our case, proven TB transmission is challenging; generally, it presents itself early after SOT, most commonly as fever, and carries a high mortality risk.

Concerning the information gaps, we should reinforce that the donor had cultures pending at the time of procurement. It should be commented that culture results are routinely checked on day 7. However, mycobacterial culture is more time-consuming, and this result is not systematically evaluated. In addition, it should be considered that the donor tests and samples are processed and held at the hospital where the donor was treated, and the organs were removed for donation. In our case, the microbiological results were detained in the laboratory hospital in which the donor was assisted. It must be stated that there was no specific protocol defining who was responsible for checking the pending test results.

Indeed, if we had known this result sooner, we could have started treatment earlier. In this regard, a compelling article [6] showed that communication gaps in reporting DDI are frequent and may occur at multiple levels, contributing to adverse outcomes among affected organ recipients. Organ donor screening for infections is currently based on donor history, physical assessment, and laboratory testing; however, circumstances such as the deviation of attention to the care of this donor and her baby, incomplete medical records, lack of training, and an insufficiently tight network for monitoring these results remain challenging and may have contributed to this catastrophic outcome.

High-risk complications of the recipient with eventual connection with the donor, such as death or sepsis within 3 months of the procedure, should be actively notified. In the reported case, if a flag for a possible DDI was detected after the first recipient's death, the others could certainly have had a better chance.

Another gap might be related to the compulsory communication of vertical TB transmission. The newborn was diagnosed with TB in the first 2 months of life. If this information had been shared earlier, screening the other transplant recipients for TB would have been possible.

Historically, many guidelines and protocols emphasize the importance of registry and the use of procedures for the safety and prevention of infectious disease transmission [38–40]. Given that, the surveillance of DDI is a strong indicator of transplant safety. Biovigilance initiatives notably in the United States and Europe have been implemented with the aim of developing national surveillance systems for cells, tissues, and organs.

To date, Brazil does not have as yet a biovigilance system (donor traceability) completely deployed. The current system works on demand and is triggered when the transplant program communicates an adverse event that may impact the recipients' survival or their quality of life. This adverse event could be an infectious disease or neoplasia that was undiscovered at procurement but diagnosed after the transplant procedure. In addition, any early posttransplant death related to infectious diseases has to be informed and registered. According to the Brazilian current legislation, graft and recipient survival is the only information for mandatory communication (along with the use of immunosuppressive drugs). Of note, these types of information may not interfere with the real-time quality of life. Finally, it should be pointed out that adverse events such as those described here are very rare but may occur in any place or situation. A more efficient reporting system will not prevent the event from occurring but may of course impact the outcome. All in all, a fully developed network that integrates transplant centers, OPO's, and laboratories is mandatory and could allow the recognition of potential hazards followed by a more rapid intervention.

This paper underscores the importance of prompt notification of any suspicious cases of infection transmitted by the donor, including TB, allowing to trace all recipients at risk in a timely manner.
